# Biofilm Responsive Zwitterionic Antimicrobial Nanoparticles to Treat Cutaneous Infection

**DOI:** 10.1021/acs.biomac.1c01274

**Published:** 2021-12-16

**Authors:** Sybil Obuobi, Anna Ngoc Phung, Kjersti Julin, Mona Johannessen, Nataša Škalko-Basnet

**Affiliations:** † Drug Transport and Delivery Research Group, Department of Pharmacy, UIT The Arctic University of Norway, Tromsø 9037, Norway; ‡ Host Microbe Interaction research group, Department of Medical Biology, UIT The Arctic University of Norway, Tromsø 9037, Norway

## Abstract

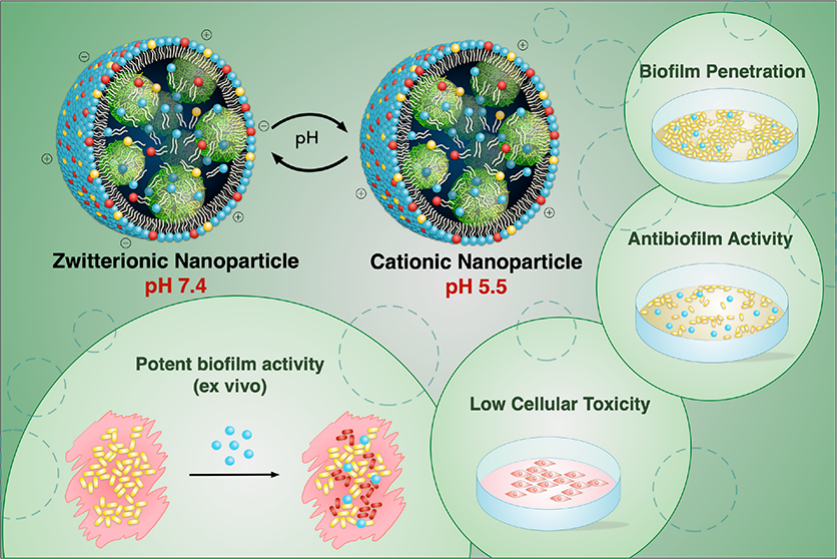

To avert the poor bioavailability of antibiotics during *S. aureus* biofilm infections, a series of zwitterionic nanoparticles containing nucleic acid nanostructures were fabricated for the delivery of vancomycin. The nanoparticles were prepared with three main lipids: (i) neutral (soy phosphatidylcholine; P), (ii) positively charged ionizable (1,2-dioleyloxy-3-dimethylaminopropane; D), and (iii) anionic (1,2-dipalmitoyl-*sn*-glycero-3-phospho((ethyl-1′,2′,3′-triazole) triethylene glycolmannose; M) or (cholesteryl hemisuccinate; C) lipids. The ratio of the anionic lipid was tuned between 0 and 10 mol %, and its impact on surface charge, size, stability, toxicity, and biofilm sensitivity was evaluated. Under biofilm mimicking conditions, the enzyme degradability (via dynamic light scattering (DLS)), antitoxin (via DLS and spectrophotometry), and antibiotic release profile was assessed. Additionally, biofilm penetration, prevention (*in vitro*), and eradication (*ex vivo*) of the vancomycin loaded formulation was investigated. Compared with the unmodified nanoparticles which exhibited the smallest size (188 nm), all three surface modified formulations showed significantly larger sizes (i.e., 222–277 nm). Under simulations of biofilm pH conditions, the mannose modified nanoparticle (PDM 90/5/5) displayed ideal charge reversal from a neutral (+1.69 ± 1.83 mV) to a cationic surface potential (+17.18 ± 2.16 mV) to improve bacteria binding and biofilm penetration. In the presence of relevant bacterial enzymes, the carrier rapidly released the DNA nanoparticles to function as an antitoxin against α-hemolysin. Controlled release of vancomycin prevented biofilm attachment and significantly reduced early stage biofilm formations within 24 h. Enhanced biocompatibility and significant *ex vivo* potency of the PDM 90/5/5 formulation was also observed. Taken together, these results emphasize the benefit of these nanocarriers as potential therapies against biofilm infections and fills the gap for multifunctional nanocarriers that prevent biofilm infections.

## 1 Introduction

Bacteria can colonize the surface of diseased tissues or medical devices to form biofilms, which has significant medical, social, and economic ramifications. Biofilms are described as communities of bacteria embedded in a protective matrix of extracellular polymeric substance (EPS), which comprise biomolecules such as lipopolysaccharides, lipids, polysaccharides, proteins, and deoxyribonucleic acid (DNA).^1^ The U.S. National Institute of Health (NIH) reports that 65% to 80% of all microbial and chronic wound infections are attributable to biofilm communities.^2^ Compared with planktonic cells, bacteria cells residing in biofilms are 100 to 1000× more resistant to antimicrobial therapy and represent the greatest obstacle to chronic wound healing.^3,4^ The poor performance of antibiotics against biofilms has been attributed to limited drug diffusion and deactivation of antibiotics (via matrix binding or an increased production of degrading enzymes which inactivate or neutralize the antibiotics).^5−7^ To avert the high mortality associated with biofilm infections, engineered nanocarriers increasingly constitute an advanced approach to improve the efficacy of antibiotics and overcome biofilm resistance.

Within this context, DNA carriers (e.g., DNA tetrahedron cages and DNA nanogels) are among the most promising candidates for antimicrobial drug delivery.^8,9^ A vast majority of DNA nanostructures have demonstrated reduced drug toxicity, controlled drug release, and enhanced antimicrobial efficacy against planktonic cells. Previous studies has also established the excellent biocompatibility, low immunogenicity, and *in vivo* stability of DNA nanoobjects.^10,11^ Because most pristine DNA nanoparticles cannot interact with microbial cells, surface functionalization is often required to achieve pathogen targeting. In fact, Hui and colleagues exploited this caveat as an antifouling strategy and demonstrated a significant reduction in bacteria cell adhesion on DNA-patterned wafers prepared with triangular DNA nanostructures.^12^ Conversely, aptamer functionalization on DNA origami nanoparticles enabled high nanostructure affinity for bacterial targets (*Bacillus subtilis* and *Escherichia coli*) compared with origami structures without the aptamers.^13^ In parallel research efforts, directed growth and assembly of silver nanoparticles was achieved using polycytosine DNA.^14^ These hybrids demonstrated antimicrobial activity because of the high affinity of cationic silver to the negatively charged bacteria cell wall. Along these lines, the surface chemistry of DNA-based nanocarriers can be modified via complexation with lipids to promote their interaction with the EPS components of biofilms. Such modifications can also enhance the responsiveness of DNA nanoobjects to the biofilm milieu. However, surface modification of nanocarriers with cationic lipids poses toxicity challenges which must be addressed.

**Scheme 1:**
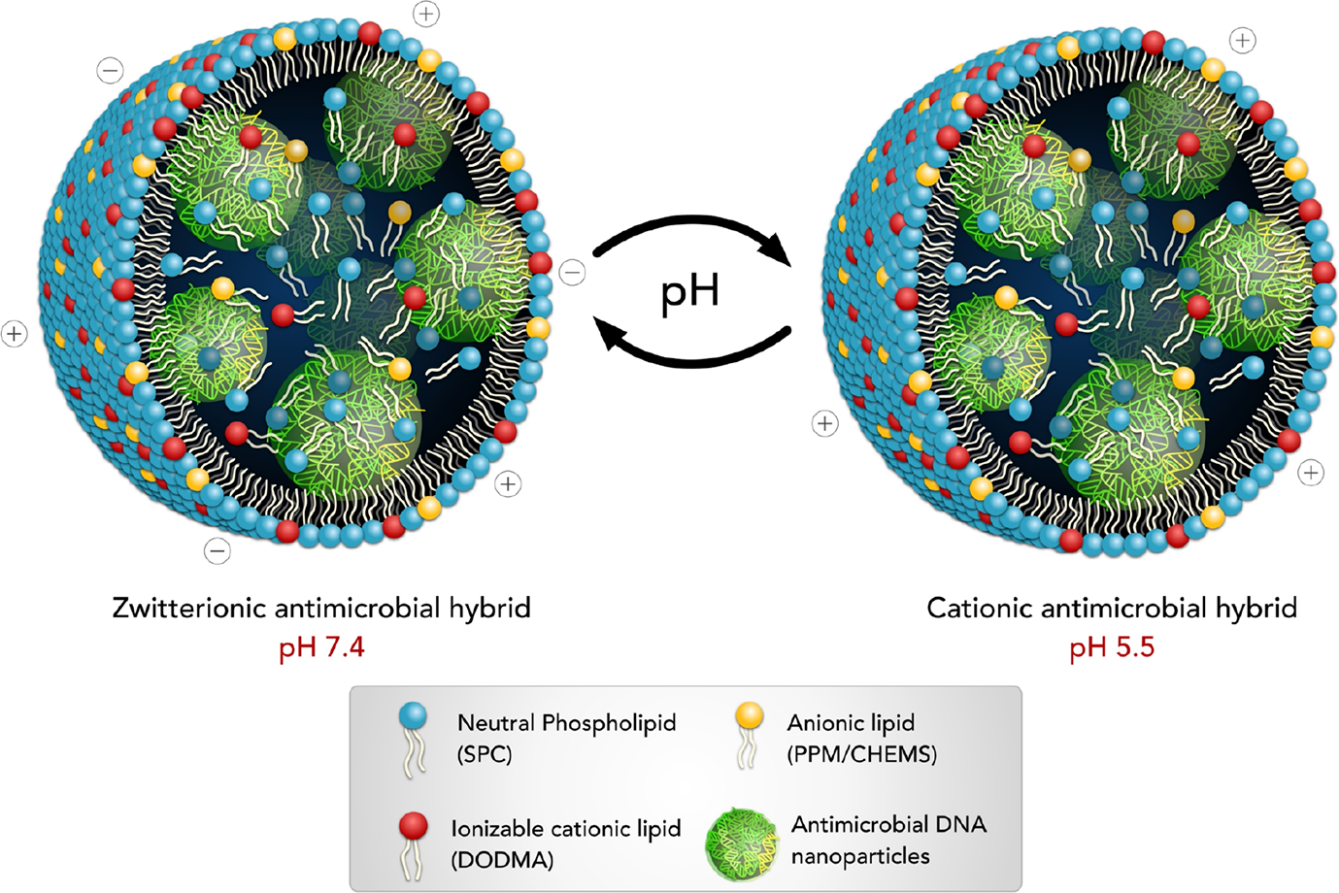
Schematic Representation of the Zwitterionic Nanoparticles and Their pH Responsiveness. Image Created by The Micro Art Illustrations

Therefore, we hypothesized that surface modification of DNA nanoparticles with pH-responsive lipids is a promising strategy to effectively deliver antimicrobials against microbial biofilms. To enhance interaction with components of the biofilm, we prepared lipid complexed DNA nanoparticles. To promote penetration through the biofilm matrix, lipid modification was achieved with positively charged ionizable (1,2-dioleyloxy-3-dimethylaminopropane), anionic (1,2-dipalmitoyl-*sn*-glycero-3-phospho((ethyl-1′,2′,3′-triazole) triethylene glycolmannose or cholesteryl hemisuccinate), and neutral lipids (soy phosphatidylcholine) (Scheme 1). Under physiological conditions, the carrier displays a neutral surface charge while under stimulations of the acidic biofilm microenvironment, protonation of the ionizable lipid leads to a positively charged system. Against preformed biofilms, the cationic nanoparticle facilely promotes rapid biofilm binding and penetration. The sensitivity of the carrier to bacterial enzymes inhibited biofilm formation at low antibiotic concentrations, and the released nucleic acid nanoparticles neutralized bacteria endotoxins. Compared with the cationic control, introduction of the anionic lipid obscured the visibility of the nanocarrier to dermal cells which significantly improved cellular viability. The translational value of the formulation against cutaneous wound infections was demonstrated in a porcine explant model where a single application of the zwitterionic formulation led to a potent reduction in the bacterial bioburden within 24 h.

## 2 Experimental Section

### 2.1 Materials

1,2-Dioleyloxy-3-dimethylaminopropane (DODMA), 1,2-dipalmitoyl-*sn*-glycero-3-phospho((ethyl-1′,2′,3′-triazole)triethylene glycolmannose (PPM), cholesteryl hemisuccinate (CHEMS), and 1,2-dioleoyl-*sn*-glycero-3-phosphoethanolamine-*N*-(lissamine rhodamine B sulfonyl) were purchased from Avanti Polar Lipids (Alabama, U.S.A.). Lipoid S100 (soy phosphatidylcholine (SPC)) was a kind gift from Lipoid GmbH (Ludwigshafen, Germany). Uranyless was purchased from Electron Microscopy Sciences (U.S.A.). Methanol was purchased from VWR International S.A.S. (Fontenay-sous- Bois, France). 3-(4,5-Dimethylthiazol-2-yl)-2,5-diphenyltetrazolium bromide (MTT), crystal violet 1%, Dulbecco Modified Eagle’s medium (DMEM) high glucose, components of encapsulation buffer (EB) (5 mM Tris-HCL, 1 mM ethylenediaminetetraacetic acid (EDTA), 10 mM MgCl2, 10 mM NaCl), α-hemolysin, lipase (from wheat germ, ∼0.1 U/mL), phosphate-buffered saline (PBS), fetal bovine serum (FBS), penicillin-streptomycin, 5× trypsin, lipoteichoic acid (LTA), and vancomycin were purchased from Sigma-Aldrich (Norway). AlexaFluor-594 labeled lipopolysaccharide (LPS) and AlexaFluor-594 labeled dextran were purchased from Thermo Fisher Scientific. Tryptic soy broth (TSB) and Luria broth agar (LA) were obtained from University Hospital of North Norway (Tromsø, Norway). All the DNA sequences were purchased from Integrated DNA Technologies (Belgium).^15^

### 2.2 Bacterial Strains and Mammalian Cells

*Staphylococcus aureus* (*S. aureus*) RN4220 containing the plasmid pCM29 encoding green fluorescent protein GFP was a kind gift from Alexander Horswhill.^16^ The plasmid was isolated and transformed into chemically competent *Escherichia coli* (*E. coli*) Top10 cells (Invitrogen) and plated out on LB agar containing 10 μg/mL chloramphenicol. The pCM29 plasmid was isolated from transformants and precipitated using pellet paint coprecipitant (Merck, Germany) and electroporated into *E. coli* IM01 cells. Plasmid DNA was isolated from transformants, precipitated, and electroporated into *S. aureus* NCTC 8325-4. Transformants were selected on TSA plates supplements with 10 μg/mL chloramphenicol. Human immortal keratinocytes (HaCaT) were purchased from CLS Cell line service GmbH (Germany) and maintained in DMEM supplemented with 10% FBS, penicillin (100 units/ml), and 100 μg/mL streptomycin in an incubator (5% CO_2_) at 37 °C.

### 2.3 Preparation of Zwitterionic Nanoparticles

The blank and the vancomycin-loaded DNA nanoparticles were prepared with slight modifications from previous reports.^15^ To prepare the lipid matrix, pure soy phosphatidylcholine (SPC) (Lipoid S100), 1,2-dioleyloxy-3-dimethylaminopropane (DODMA), and 1,2-dipalmitoyl-*sn*-glycero-3-phospho((ethyl-1′,2′,3′-triazole)triethylene glycolmannose (PPM) or cholesteryl hemisuccinate (CHEMS) were dissolved in a round-bottom flask with methanol. Lipid films were prepared using the thin film hydration method and rehydrated with the DNA nanoparticles to form the zwitterionic nanoparticles. The solutions were extruded through polycarbonate membranes with pore sizes of 800, 400, and 200 nm. The formulations were then stored at 4 °C until needed. Zwitterionic nanoparticles comprising different lipid mole percentages were prepared as shown in Table 1.

**Table 1:** Lipid Composition of the Zwitterionic Nanoparticles^a^

formulation	**P**(mol %)	**D** (mol %)	**M** (mol %)	**C**(mol %)
PD (95/5)	95	5	-	-
PDM (92.5/5/2.5)	92.5	5	2.5	-
PDM (90/5/5)	90	5	5	-
PDC (92.5/5/2.5)	92.5	5	-	2.5
PDC (90/5/5)	90	5	-	5
PDC (85/5/10)	85	5	-	10

a **P**: *Soy phosphatidylcholine* (SPC) ; **D**: *1.2-Dioleyloxy-3-dimethylaminopropane* (DODMA) ; **M**: *1,2-dipalmitoyl-sn-glycero-3-phospho((ethyl-1*′,2′,3′*-triazole)triethylene glycolmannose* (PPM) ; **C**: *Cholesteryl hemisuccinate* (CHEMS).

### 2.4 Nanoparticle Characterization

The hydrodynamic diameter (size), polydispersity index (PDI), and zeta potential of the formulations were determined using the Zetasizer (Nano-z, Malvern instruments) via dynamic light scattering (DLS) technique. For size measurements, the nanoparticles were diluted 100× in 1× EB buffer prior to use. However, zeta potential measurements were performed on nanoparticles that were diluted 50× in tap water. The measurements were performed at room temperature, and the average reading was recorded as mean ± standard deviation (SD). The morphology of the nanoparticles was investigated by transmission electron microscopy (TEM). Briefly, dilutions of the formulation were stained with UranyLess and dried for 20 min. The samples were imaged using TEM (HT7800, Hitachi).

### 2.5 Biofilm Sensitivity and Binding

To study the interactions between the formulation and components of the biofilm matrix, AlexaFluor 594 labeled lipopolysaccharide (LPS), lipoteichoic acid (LTA), and AlexaFluor 594 dextran were chosen as models of the EPS. Changes in the fluorescence intensity of LPS in the presence of the zwitterionic nanoparticles was assessed. Prior to experiments, a reference fluorescence curve of the LPS (0.25, 0.5, 1, and 2 μg/mL) was obtained using the microplate reader (Spark, Tecan). Thereafter, 50× dilution of the nanoparticle formulation was prepared and mixed with the LPS (final concentration of 4 μg/mL). Then 50 μL of the mixture was transferred into a costar black 96-well plate for fluorescence measurements. For the negative and positive controls, 1× EB buffer or DNA nanogels were used, respectively. For experiments with LTA, fluorescently labeled PDM 90/5/5 (PDM^Rho^) was prepared with the addition of 1,2-dioleoyl-*sn*-glycero-3-phosphoethanolamine-*N*-(lissamine rhodamine B sulfonyl). PDM^Rho^ 90/5/5 was diluted 100× and mixed with 20 μg/mL LTA. Subsequently, 70 μL of the mixture was transferred into a costar black 96-well plate for fluorescence measurements. PDM^Rho^ 90/5/5 alone and LTA alone were used as controls. Similarly, the interaction between dextran and the formulation was assessed by recording the changes in fluorescence. A reference fluorescence peak curve of dextran was also obtained between 0.25, 0.5, 1, and 2 μM. A final concentration of 1 μM dextran was added to serial dilutions of the formulation (25×–1600×), whereas 1× EB buffer serves as the negative control. Triplicate volumes of the samples were added in a costar black 96-well plate, and the fluorescence intensity was measured in the plate reader (Spark, Tecan). All results are reported as means.

To assess the sensitivity of the formulation to lipase, changes in the size of the nanoparticles were analyzed using the Zetasizer (Nano-Z, Malvern instruments) in the presence of varying concentrations of lipase (0.3, 0.5, 1, 2, 4, and 8 mg/mL). The size of the samples were measured at 0 h, 1 h, 2 h, 5 h and 24 h. Three replicates were obtained for each measurement. To study the interactions with α-hemolysin, changes in the absorbance peak of ssDNA and the size of the DNA nanoparticles was studied. Briefly, varying concentrations of the toxin was incubated with the vancomycin loaded DNA nanogels in 1× EB buffer. After 10 min, the size of the nanoparticle was measured using the Zetasizer (Nano-Z, Malvern instruments). Changes in the absorbance of ssDNA (4 μM) in the presence of different concentrations of the toxin (1, 2, 4, and 8 μg/mL) was evaluated.

### 2.6 Determination of Percentage Entrapment Efficiency

The entrapment efficiency (EE %) was evaluated using the dialysis bag method. 500 μL of the formulation was dialyzed (mw 12–14 kDa) in a beaker containing 50 mL of 1× EB buffer. After 4 h, the nanoparticles were disrupted with methanol (10× dilution). Removal of the DNA nanostructures was done using centrifugal filter units, and the samples were centrifuged at 13 000 rpm for 2 min. The absorbance of the flow-through solution containing vancomycin was then measured using the UV-spectrophotometer. The entrapment efficiency of vancomycin was then calculated on the basis of the absorbance values using a pre- obtained calibration curve. The following equation was used to calculate the entrapment efficiency where *C*_encap_ refers to the drug concentration in the dialyzed formulation and *C*_total_ refers to the drug concentration in the undialyzed samples:
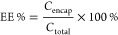


### 2.7 *In Vitro* Drug Release

The *in vitro* release of vancomycin was assessed using a multistation Franz diffusion cell system. Briefly, the system was heated to 35 °C, and the 5 mL receptor chambers were filled with PBS at pH 7.4 under constant stirring. Prior to use, fitted cellophane membranes were cut out and submerged into deionized water for 5–10 min, and then the membranes were sandwiched between the receptor chamber and the donor chamber. Thereafter, 700 μL of the formulation or free drug was loaded into the donor cell. To examine the influence of bacteria enzymes on the drug release profile of the formulation, lipase (final concentration of 1, 4, or 8 mg/mL) was added to the donor cell, and then the chamber was sealed with rubber plug and parafilm.

Samples of the released antibiotic were taken, and the receptor chambers were refilled with 500 μL of buffer to ensure a continuous sink condition. The amount of drug released at each time point was quantified via absorbance readings and reported as the percentage mean ± SD from duplicate readings in reference to the total amount of drug loaded in the formulation prior to the start of the experiment. The following equation was used to calculate the cumulative drug release percentage:
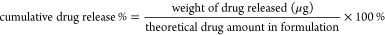


### 2.8 Biofilm Studies

#### Biofilm Inhibition

To evaluate the ability of the nanoparticles to inhibit biofilm formation, a *S. aureus* strain containing a plasmid constitutively expressing green fluorescent protein (GFP) (NCTC 8325 pCM29GFP) was grown in the presence or absence of the nanoparticles. For the bacterial work, 10 mL of TSB (supplemented with chloramphenicol 10 μg/mL) was inoculated with the bacteria and incubated overnight (37 °C, 100 rpm). The optical density (OD) of the overnight culture was then adjusted to 0.07 (10^8^ colony forming units (CFU)/mL). The exact concentration of vancomycin in the formulation was determined via absorbance readings obtained on the UV-spectrophotometer. Serial dilutions of the formulation were prepared in TSB growth medium to give a final desired concentration range of vancomycin from 0.5 to 32 μg/mL. To grow the biofilms, 12.5% w/v glucose stock and bacteria was added to the growth media to give a final concentration of 1% w/v glucose and 10^6^ CFU respectively. Aliquots of 200 μL were separately loaded in triplicates into the 96- well plate and incubated for 24 h at 37 °C. As a control, 1× EB buffer was used. After 24 h, the biofilm growth solution was replaced with fresh media, and the fluorescence reading was taken using a microplate reader (Spark, Tecan) at excitation and emission wavelengths of 480 and 530 nm, respectively.

#### Biofilm Eradication Experiments

Biofilms were developed for 6 h, and the growth media was gently discarded and replaced with 100 μL of growth media with the formulations to give a concentration of 1, 5, 10, and 50 μg/mL. The 96-well plate was then incubated at 37 °C. After 24 h, the biofilms were quantified via fluorescent changes. For crystal violet staining, 125 μL of 0.1% w/v crystal violet was added to the biofilms and stained for 10 min. The solutions were then discarded, and the wells were washed with filtered tap water to remove the excess dye. Photographic images were then taken using a digital camera. To solubilize the dye, 200 μL of dimethyl sulfoxide (DMSO) (Sigma) was added into each well. Absorbance readings were obtained at 590 nm using a microplate reader (Spark, Tecan). The experiment was carried out in triplicates.

#### Biofilm Penetration Assay

The ability of the zwitterionic nanoparticles to bind and penetrate *S. aureus* biofilms was investigated using confocal laser scanning microscopy (CLSM). GFP-expressing *S. aureus* biofilms were grown as previously described, in 8-well chambers for 24 h. Fluorescently labeled PDM 90/5/5 formulations (PDM^Rho^) was prepared as previously described. After growing the biofilms for 24 h, the growth solution was discarded, and the biofilms were treated with 300 μL of 50 μg/mL PDM^Rho^ 90/5/5 formulation at pH 5.5 for a duration of 30 or 120 min. Prior to imaging, the biofilms were washed with sterile water to remove unbound nanoparticles on the biofilm surface. Thereafter, the biofilms were observed under CLSM with excitation and emission wavelengths for GFP (480 and 530 nm) and rhodamine B (560 and 583 nm) respectively.

### 2.9 Cytotoxicity of Zwitterionic Nanoparticles

The cell toxicity of the optimized formulation was assessed using human immortal keratinocytes, HaCaT cells. The cells were cultured in cell culture flasks with Dulbecco’s Modified Eagle’s Medium (DMEM) high glucose (supplemented with 10% w/v fetal bovine serum (FBS) and 1% penicillin-streptomycin). At 80% confluency, the cell monolayer was washed twice with 10 mL of phosphate-buffered saline (PBS). The PBS was discarded, then 3–4 mL of PBS + EDTA (0.25 mM) was added to remove cell to cell adhesion, and the flask was incubated for 10 min. Thereafter, 1 mL of 0.25% trypsin was added and further incubated for 2 min to detach the cells. The cell solution was then added to 7 mL of fresh DMEM, and the solution pipetted to separate the cells. The cell density was measured using a hand-held automated cell counter device. Afterward, 200 μL of the cell solution was seeded at a cell density of 6000 cells per well in a 96-well plate. The plates were incubated for 24 h at 37 °C. The cytotoxicity of the formulation was determined at 1, 5, 10, and 50 μg/mL final vancomycin concentrations. The exact concentration of vancomycin in the formulation was determined using a UV-spectrophotometer prior to cytotoxicity assays. Wells containing only DMEM solution served as a negative control, and free vancomycin served as a positive control. The cytotoxicity of the formulation was determined by the 3-(4.5- dimethylthiazol-2-yl)-2.5-diphenyltetrazolium bromide (MTT) assay. Briefly, 200 μL DMEM containing MTT (0.5 mg/mL) was added to each well and incubated for 2–4 h. After incubation, the MTT solution was removed, and 100 μL of DMSO was added into each well to dissolve the formazan crystals. Quantification of formazan was done using the microplate reader (Spark, Tecan).

### 2.10 *Ex Vivo* Pig Skin Biofilm Eradication Model

The efficacy of the zwitterionic nanoparticles was further assessed using an *ex vivo* pig skin model with slight modifications.^17,18^ Sections of pig ear (5 mm) were cut using a circular biopsy punch, transferred into a 24-well plate, and thoroughly washed three times with sterile water. The skin sections were disinfected using chloramphenicol (10 μg/mL for 1 h) and 70% ethanol (for 20 min). After the samples were washed with PBS and dried for 1 h, the skin sections were transferred with the skin side up to a 96-well plate containing 150 μL of 0.5% solidified agar (Sigma) to ensure hydration of the skin. The skins were allowed to dry for 15 min prior to addition of 20 μL of GFP-expressing *S. aureus* (equivalent to 10^6^ CFU). The plate was incubated at 37 °C for 24 h to establish the biofilms. Then, 50 μL of the formulation was added to the skin sections, and the plate was again incubated for an additional 24 h. For the negative control, 1× EB buffer was applied to the skin sections. Uninfected skin sections were also prepared to adjust the fluorescence baseline. PBS was added to the empty wells to keep the skins well hydrated. Postincubation, the sections were transferred to a black 96-well plate, and the fluorescence changes were measured on microplate reader (Spark, Tecan). The experiments were performed in triplicate.

### 2.11 Statistical Analysis

All experimental data was analyzed using the GraphPad Prism 8 (La Jolla, CA). To assess the statistical significance of the experimental groups to the control, the two-sample student *t* test and one-way ANOVA analysis were performed. For multiple comparisons, the Dunnett’s test was used. The results were reported as statistically significant for *p* <. 05.

## 3 Results and Discussion

### 3.1 Effect of PPM and CHEMS on Zeta Potential

A series of zwitterionic nanoparticles were prepared with varying lipid compositions as illustrated in Table 1. The lipid matrix of the formulations comprised DODMA (1.2-Dioleyloxy-3-dimethylaminopropane), SPC (soy phosphatidylcholine), PPM (1,2-dipalmitoyl-*sn*-glycero-3-phospho((ethyl-1′,2′,3′-triazole)triethylene glycolmannose), and CHEMS (cholesteryl hemisuccinate) (Scheme 1). First, the blank DNA nanoparticles were complexed with approximately 7.5–7.8 mg/mL of the lipids, and the zeta potential was examined at different pH conditions. The ratio of CHEMS or PPM was tuned between 0 and 10 mol % to ascertain the optimal ratio of the anionic lipids.

**Figure 1:**
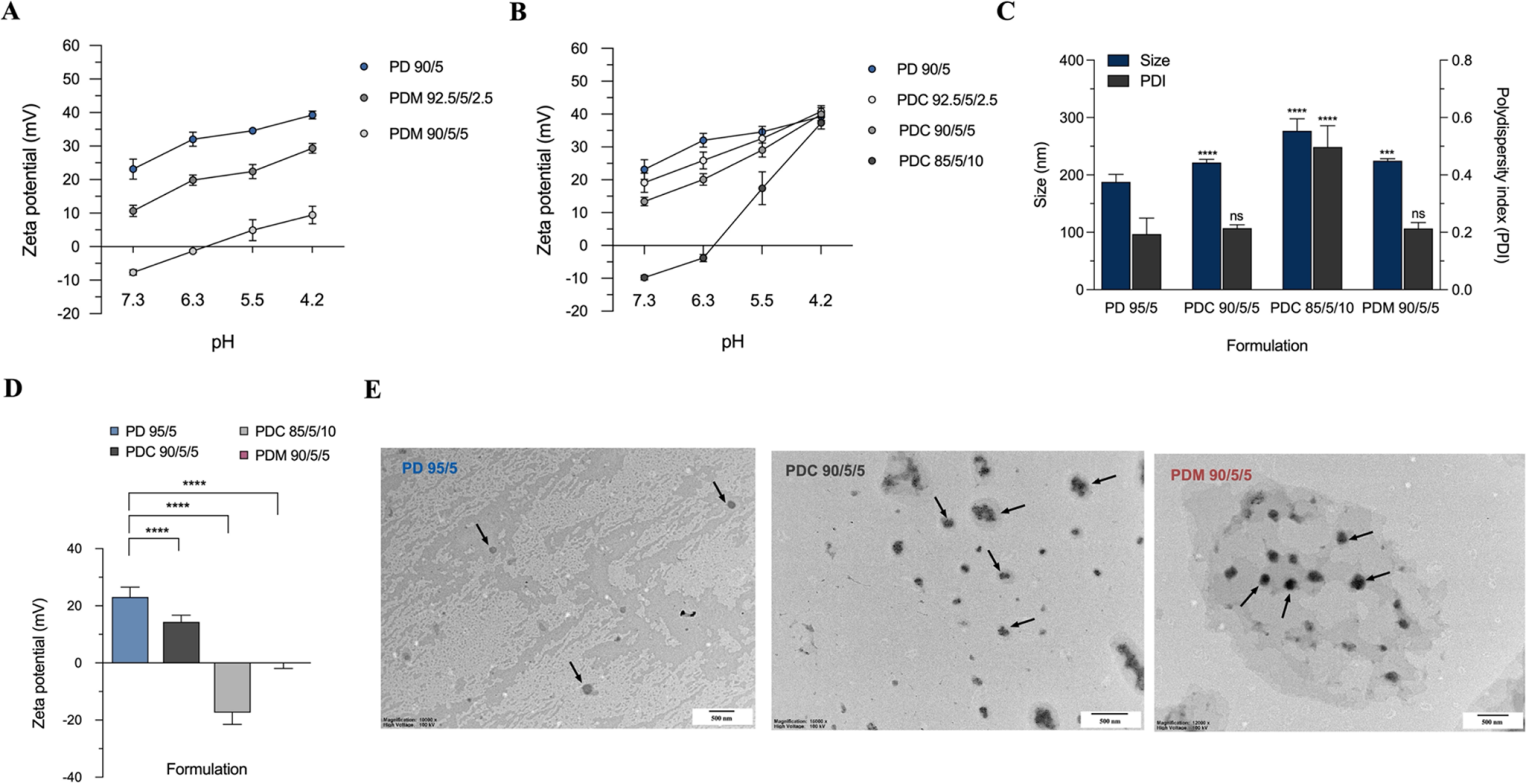
Characterization of the zwitterionic nanoparticles using DLS and TEM imaging. (A) Effect of PPM on zeta potential of the blank PDM formulations at different pH conditions. (B) Effect of CHEMS on zeta potential of the blank PDC formulations at different pH conditions. (C) Particle size and PDI measurements of the different antimicrobial nanoparticles. (D) Zeta potential measurements of the different antimicrobial nanoparticles at pH 7.4 (all values based on mean ± SD; *n* = 3). (E) Morphology of the PD 95/5, PDC 90/5/5, and PDM 90/5/5 formulation (scale bar = 500 nm).

As shown in Figure 1A, the blank PD 95/5 formulation demonstrated the most positive zeta potential (+23.13 ± 2.99 mV at pH 7.3 and +39.28 ± 1.17 mV at pH 4.2) due to the highly cationic nature of the ionizable lipid, DODMA. In keeping the mole percentage of DODMA constant, we reduced the ratio of the neutral SPC lipid since it played a negligible role on the surface charge and incorporated the anionic lipids to prepare the PDM (PPM) or PDC (CHEMS) formulations. At the lowest concentration tested (i.e., 2.5 mol %), the PDM 92.5/5/2.5 formulation presented a positive zeta potential of +10.64 ± 1.67 mV at pH 7.3 and +29.32 ± 1.45 mV at acidic pH (pH 4.2) (Figure 1A). Since this system was not optimal, we increased the concentration of PPM to 5 mol %. Zeta potential measurements of PDM 90/5/5 revealed a close to neutral surface potential of −7.69 ± 0.79 mV at pH 7.3 with a sharp reversal to a cationic value of +9.42 ± 2.64 mV at acidic pH. This reduced zeta potential is attributed to the increased concentration of the anionic PPM which neutralizes DODMA.

In reciprocating the above-mentioned mole percentages, the PDC 92.5/5/2.5 and PDC 90/5/5 formulations presented a positive zeta potential of +19.12 ± 2.92 mV and +13.39 ± 1.27 mV, respectively, at physiological pH (Figure 1B). At acidic pH (4.2), a significant increase in the zeta potential to +40.76 ± 1.83 mV (PDC 92.5/2.5/2.5) and +39.93 ± 2.01 mV (PDC 90/5/5) was observed. Compared to the PDM 90/5/5, the PDC 90/5/5 formulation exhibited a more drastic switch in charge at acidic pH (i.e., a change of ∼+ 27 mV was seen for the PDC formulations compared with a change of ∼+ 17 mV for the PDM formulations). This is attributable to the ionizable cationic DODMA in corporation with CHEMS, whose surface is highly affected by pH. Seeking to identify the concentration of CHEMS needed to completely neutralize the zeta potential of the PDC formulations, PDC 85/5/10 was prepared, which showed a more negative zeta potential of −9.78 ± 0.69 mV at pH 7.3. Despite the increment of the mole percentage of CHEMS, the PDC 85/5/10 formulation also exhibited a highly cationic zeta potential of +37.3 ± 1.81 mV at pH 4.5 (Figure 1B). This can be attributed to the pH-dependent reversal of CHEMS from a lamellar phase to a hexagonal phase. Hafez and Cullis described this polymorphism and demonstrated that CHEMS adopts the hexagonal phase at pH values below the PK of the succinate headgroup.^19^ Unlike the PDC formulation, the PDM retains its lamellar phase wherein the exposed anionic headgroup neutralizes DODMA even at acidic pH. In both cases, however, we observed a strong correlation between the mol % of the anionic lipid to the surface charge at physiological pH. This direct relationship was also observed when cationic protamine–DNA complexes were mixed at different ratios with preformed CHEMS/DOPE liposomes.^20^ Thereafter, to compare the physicochemical properties of the antimicrobial nanoparticles, formulations with similar mole percentage were chosen (i.e., PDC 90/5/5 and PDM 90/5/5). However, since the PDC 90/5/5 formulation presented cationic values at both physiological and acidic pH, the anionic PDC 85/5/10 formulation was incorporated as a control.

### 3.2 Physicochemical Characterization of the Antimicrobial Formulations

The vancomycin-loaded formulations were prepared and characterized for their size, PDI, zeta potential, and morphology. As shown in Figure 1C, in the absence of CHEMS or PPM, the size of the PD 95/5 nanoparticles was 187.77 ± 13.10 nm. Following incorporation of the anionic lipids, a significant increase in size to 221.67 ± 5.51 nm and 224.57 ± 3.69 nm was observed for the PDC 90/5/5 and PDM 90/5/5 formulations, respectively. This observation can be attributed to the electrostatic repulsion between the polyanionic DNA and the anionic lipids in the PDM and PDC formulations. In agreement with this, the PDC 85/5/10 formulation displayed an even larger size of 276.97 ± 20.80 nm. PDI analysis revealed a homogeneous and narrow size distribution of 0.19 ± 0.06, 0.21 ± 0.01, and 0.21 ± 0.02 for the PD 95/5, PDC 90/5/5, and PDM 90/5/5 formulations, respectively (Figure 1C). However, the PDC 85/5/10 formulation presented a higher PDI of 0.50 ± 0.07.

Zeta potential measurements revealed a cationic charge of +23.09 mV ± 3.46 mV for the PD 95/5 formulation. For the PDC 90/5/5 formulation, a significant reduction to +14.39 ± 2.30 mV was observed (Figure 1D). A flip to a highly negative surface potential was observed for the PDC 85/5/10 (−17.36 mV ± 4.13 mV) formulation. The negative surface charge of the PDC 85/5/10 further corroborates the high PDI, which is due to the enhanced electrostatic repulsion and lower entrapment of the DNA nanoparticles. Major limitations of DNA delivery via cationic lipid nanoparticles include aggregation, toxicity, and low release. To address these challenges, Fillion and colleagues defined conditions to encapsulate antisense oligonucleotides in anionic liposomes using encapsulation solutions with monovalent salts.^21^ In another study, the effect of divalent ions (i.e., Ca^2+^) on the interactions between DNA and anionic multilamellar vesicles was investigated.^22^ Because of the presence of electrostatic repulsion, no interactions were observed in the absence of calcium ions. The antimicrobial PDM 90/5/5 formulation displayed an ideal neutral surface potential of −0.02 ± 1.89 mV because of neutralization of the charged head groups. To further correlate the DLS measurements, the morphology of the nanoparticles was investigated using transmission electron microscopy (TEM). As indicated in Figure 1E, all three formulations displayed a spherical shape. Visually, the PD 95/5 formulation revealed a smaller size than the PDM 90/5/5 and the PDC 90/5/5 formulation and agrees with the DLS analysis.

**Figure 2:**
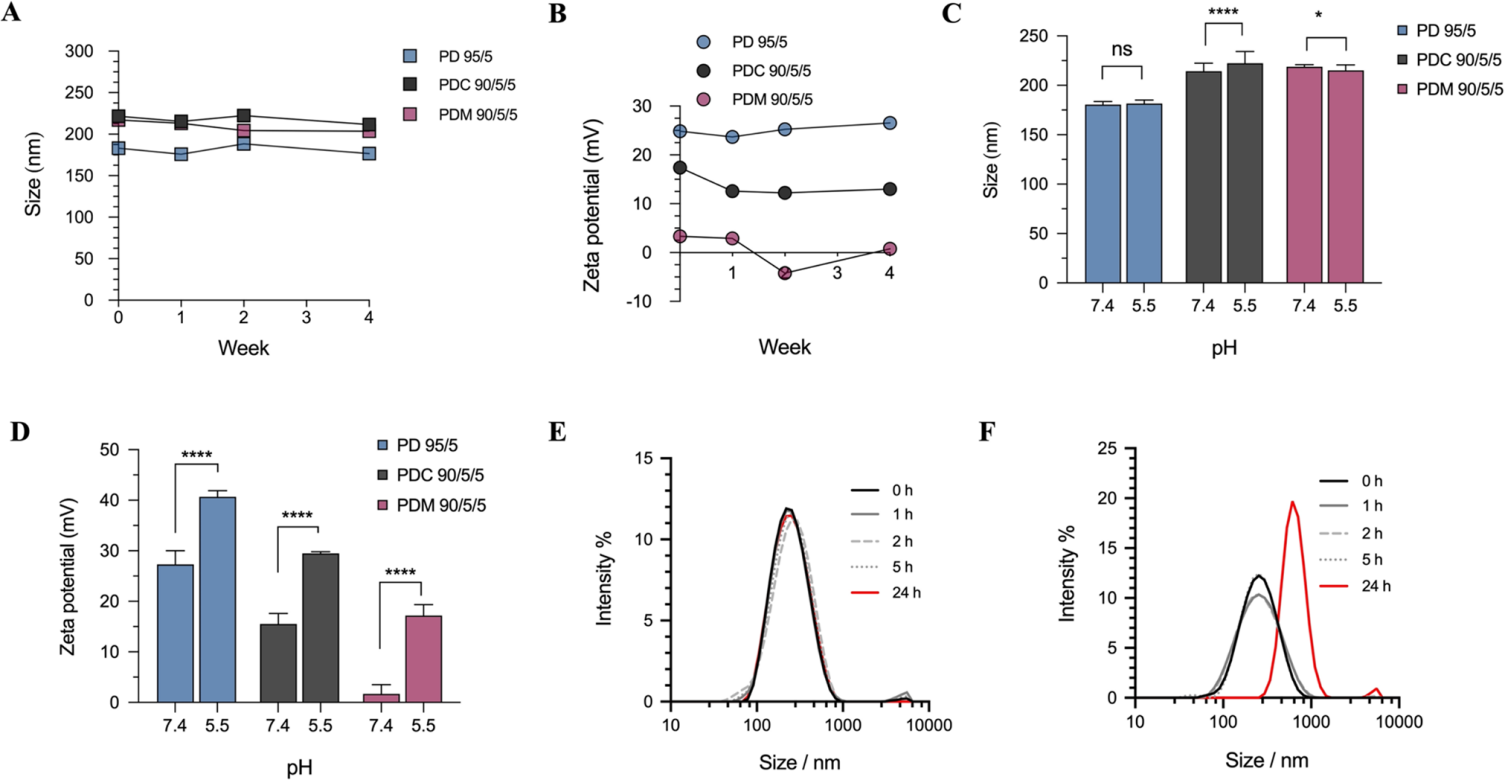
Effect of pH, time, and storage conditions on the nanoparticles. (A) Effect of storage on the size of the different formulations. (B) Effect of storage on the zeta potential of the different formulations. (C) Effect of pH on the size of the different formulations (*n* = 2). (D) Effect of pH on the zeta potential of the different formulations (*n* = 2). (E) Size distribution of the PDM 90/5/5 formulation at pH 5.2 over 24 h. (F) Size distribution of the PDC 90/5/5 formulation at pH 5.2 over 24 h.

Nanoparticle aggregation is detrimental and can lead to the leakage of entrapped nucleic acid nanoparticles and/or the cargo.^23^ Because formulations with zeta potentials greater than −30 mV or +30 mV have been shown to possess better colloidal stability,^24^ changes in size and zeta potential of the formulations were monitored to establish the absence of aggregation. As shown in Figure 2A, no relevant increase in the size of the nanoparticles was observed after 4 weeks, indicating the absence of aggregates within this period. Zeta potential measurements of the formulations revealed a drop in zeta potential for the PDC 90/5/5 formulations (Figure 2B) from +17.40 ± 0.17 mV to +13.03 ± 0.32 mV after 4 weeks. Conversely, the PDM 90/5/5 formulations maintained a neutral charge of +0.77 ± 0.26 mV while the PD 95/5 formulation remained highly cationic (+26.57 ± 0.25 mV). However, formulations with high cationic charges have also been reported to possess higher toxicity.^25,26^ Therefore, the subtle positive charge of the PDM 90/5/5 observed at low pH conditions can minimize the toxicity of the PD 95/5 and PDC 90/5/5 formulations. Given the absence of aggregation, a stable neutral charge and reversible zwitterionic properties, the PDM 90/5/5 formulation was deemed most suitable for our application.

### 3.3 Surface-Adaptive Properties of the Antimicrobial Formulations and Affinity to Bacteria

To ensure that antibiotic-loading did not influence the surface-adaptive properties, we compared the size and zeta potential of the vancomycin-loaded formulations at pH 7.4 and 5.5. As shown in Figure 2C, no significant changes in size were observed for the PD 95/5. A slight reduction in size was seen for the PDM 90/5/5 formulations (from 218.82 ± 2.09 nm to 215.00 ± 5.80 nm).

However, the PDC 90/5/5 formulation showed significant increment from 214.32 ± 8.15 nm at pH 7.4 nm to 222.37 ± 11.89 nm at pH 5.5. Correspondingly, an altered intensity and size distribution at acidic pH after 24 h (Figure 2F) was observed for the PDC 90/5/5, whereas the PDM 90/5/5 formulation exhibited no change in size distribution (Figure 2E). This observed change for the PDC formulation is potentially due to the weakened interactions between the CHEMS and DODMA, which can promote fusion and destabilization of the nanocarrier to cause aggregation. Similar findings were observed by Sudimack and colleagues, when oleyl alcohol liposomes composed of CHEMS, egg phosphatidylcholine (PC), and Tween-80 were incubated at pH 5.0.^27^ The authors observed a time- and pH-dependent increase in particle size up to 15-fold. The antimicrobial formulations exhibited zeta potentials of +27.30 ± 2.70 mV (PD 95/5), +15.53 ± 2.06 mV (PDC 90/5/5), and +1.69 ± 1.83 mV (PDM 90/5/5) at physiological pH (Figure 2D). At acidic pH, a switch to +40.68 ± 1.20 mV (PD 95/5), +29.47 ± 0.36 mV (PDC 90/5/5), and +17.18 ± 2.16 mV (PDM 90/5/5) was observed (Figure 2D), which demonstrates the pH responsiveness of the antimicrobial nanoparticles.

### 3.4 Effect of Biofilm-Mimicking Conditions on Formulation Properties

Next, to demonstrate the sensitivity of the antimicrobial PDM 90/5/5 formulation to the biofilm microenvironment, we investigated its interaction with two components of the matrix i.e., glycolipids and polysaccharides. We first studied the interaction between PDM 90/5/5 and LTA or LPS, bacterial glycolipids relevant in the establishment of microbial biofilms. Several bacterial species rely on LPS to alter their surface attachment, transition to sessile growth and colony morphology.^28^ Sorroche and coauthors reported this phenomenon by using mutants with defective LPS and showed that these mutants exhibited reduced biofilm formation and an altered biofilm architecture compared with the wild-type strain.^29^ Additionally, Coulon and colleagues provided evidence of LPS-like materials present in the biofilm matrix of *Pseudomonas aeruginosa* (*P. aeruginosa*).^30^ In the context of biofilm formation, membrane vesicles that bleb from the outer membrane of Gram-negative bacteria have been proposed to provide large portions of LPS in the biofilm matrix.^31^ Similarly, extracellular teichoic acids (TAs) have been described as an important and permanent component of the biofilm matrix in *S. aureus*.^32^ Using clinical isolates, TAs were always found in the extracellular matrix of *S. aureus* biofilm.^33^ Kogan and colleagues attributed this to the release of TAs from the cell surface into the extracellular space to become a part of the matrix.^34^

**Figure 3:**
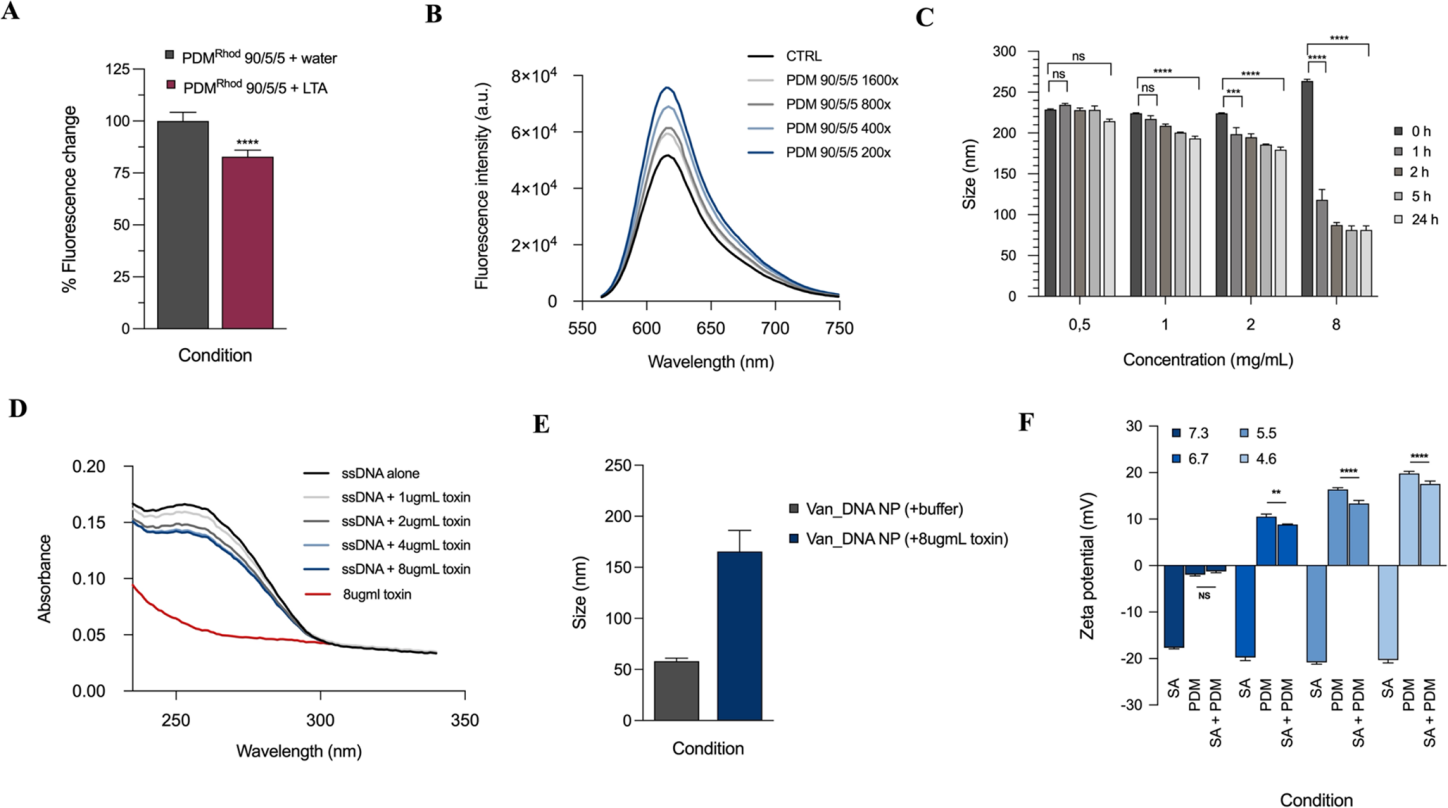
Effect of biofilm matrix components and extracellular enzymes. (A) Effect of LTA on fluorescence intensity of PDM^Rhod^ 90/5/5 at pH 5.5. (B) Effect of Alexa 594 dextran on fluorescence intensity of the PDM 90/5/5 formulation. (C) Effect of lipase on the size of the PDM 90/5/5 formulations. (D) Effect of α-hemolysin on the absorbance peak of ssDNA. (E) Effect of α-hemolysin on the size of vancomycin-loaded DNA nanogel. (F) Effect of pH on *S. aureus* interaction with the PDM 90/5/5 formulation.

Given the relevance of LPS and TAs in biofilm communities, we studied the binding affinity of the optimized PDM 90/5/5 to both bacterial components. First, changes in the fluorescence intensity of rhodamine labeled PDM 90/5/5 (PDM^Rho^ 90/5/5) was monitored in the presence of lipoteichoic acid (LTA) using fluorescence spectroscopy. PDM^Rho^ 90/5/5 was prepared by adding 1,2-dioleoyl-*sn*-glycero-3-phosphoethanolamine-*N*-(lissamine rhodamine B sulfonyl) lipid to the formulation. The intensity of the formulation was then measured in a black 96-well plate as the baseline fluorescence reading. After adding an equal volume of LTA, the percentage change in intensity was calculated. As shown in Figure 3A, a significant reduction in the fluorescence intensity (17.21 ± 3.11%) of PDM^Rho^ 90/5/5 was observed which confirms the binding affinity of LTA to the formulation. Because 1,2-dioleoyl-*sn*-glycero-3-phosphoethanolamine-*N*-(lissamine rhodamine B sulfonyl) is a headgroup labeled phospholipid, binding of LTA to the surface shields the exposed fluorophore and accounts for the reduction in the fluorescence intensity of the formulation.

Physicochemical similarities have been observed between LTA and LPS, and both biopolymers display a net negative charge.^35^ In fact, the phosphate rich molecules on LTA provide a “continuum of negative” charge that drive their interactions with cationic materials via electrostatic interactions.^36,37^ Therefore, we also studied the binding of the formulation to AlexaFluor 594 labeled LPS. To understand the effect of the surface chemistries on the observed binding affinity, PD 95/5 and PDC 90/5/5 formulations were included in the study as controls. As shown in Figure S3A, the PD 95/5 formulation showed the greatest fluorescence change 142.82 ± 3.35%. The PDC 90/5/5 and the PDM 90/5/5 formulations recorded fluorescence changes of 133.83 ± 1.68% (*p* < 0.05) and 103.94 ± 2.18% (*p* < 0.0001), respectively, which were significantly lower. We directly correlate this observation with the zeta potential measurements (PD > PDC 90/5/5 > PDM 90/5/5) at physiological pH and therefore postulate that the observed variations are highly dependent on electrostatic interactions. To confirm this, changes in fluorescence intensity was measured at acidic pH (pH 5.5). As shown in Figure S3B, no significant difference was observed between the PD 95/5 (101.54 ± 1.85%) and PDC 90/5/5 (93.43 ± 1.92%) formulation. This can be attributed to the highly cationic surface potential of these two formulations at pH 5.5. Conversely, the PDM 90/5/5 formulation showed a fluorescence increase of 58.14 ± 5.05% which was significantly lower that both the PD 95/5 and PDC 90/5/5 formulations and confirms our assessment. The relatively lower interactions of the formulation at acidic pH with LPS can be attributed to structural modifications of LPS under mildly acidic conditions due to hydrolysis as previously described in literature.^38,39^

Next, we investigated the binding affinity of the optimized PDM 90/5/5 formulation to polysaccharide components of the biofilm matrix using dextran as our model. Dextran is a well-known polymer that can be found in the EPS matrix of biofilms.^40^ It is believed that extracellular polysaccharides found in dental plagues (from cariogenic bacteria) were dextran-like polymers and contribute to the development of dental plagues. Moreover, given its chemical simplicity, it is a useful model^41^ to study nanoparticle interactions with the EPS matrix. By monitoring changes in fluorescence intensity, the interactions between Alexa 594 labeled dextran and the optimized formulation was studied. As shown in Figure 3B, in the absence of PDM 90/5/5, the measured fluorescence intensity of dextran was 50534.67 arbitrary units (a.u.). In presence of PDM 90/5/5, a concentration-dependent increase in the fluorescence intensity of fluorescently labeled dextran was observed. For instance, formulations diluted 1600× showed a smaller (14.80 ± 6.45%) increase in the fluorescence intensity compared with the 200× diluted formulation (46.41 ± 7.19%). Overall, the observed changes in the fluorescence intensity correlate the binding of the PDM 90/5/5 formulations to dextran.

Bacterial lipases are valuable extracellular enzymes produced by several bacterial species (e.g., *P. aeruginosa*, *Pseudomonas fluorescens*, *Bacillus subtilis*).^42^ These enzymes commonly known as triacylglycerol acylhydrolase catalyze the hydrolysis of lipids (e.g., triglycerides, acylglycerols, and carboxylesterases) by attacking ester bonds present on the lipid chain.^43^ Given the susceptibility of lipid-based carriers to lipolysis, antimicrobial formulations with controlled carrier degradation can be designed to stimulate drug release for the prevention and eradication of biofilms. For instance, Chen and co-workers recently developed lipase-sensitive micelles for the simultaneous release of azithromycin.^44^ Amphiphilic block copolymers of octadecylamino-terminated polyaspartamide were conjugated with azithromycin for micelle fabrication. The authors demonstrated that the grafted drug was released in response to lipases to attack bacteria and destroy the biofilms. Thus, to investigate the sensitivity of the optimized zwitterionic formulation to lipolysis, changes in the size of the PDM 90/5/5 formulation was monitored over 24 h in the presence of varying concentrations of lipase. As shown in Figure 3C, a dose- and time-dependent degradation of the carrier was observed following exposure to lipase. For instance, after exposure to 1 mg/mL lipase, a change in the size of the PDM 90/5/5 was observed from 224.27 ± 1.08 nm (at 1 h) to 193.5 ± 2.7 nm (after 24 h). Upon exposure to higher concentrations of lipase (8 mg/mL), a time-dependent degradation was observed only up until 2 h. No change in the size of the carrier was observed at 5 and 24 h. We attribute this observation to the complete degradation of the lipids at lipase concentrations of 8 mg/mL. These results further imply the sensitivity of the formulation to degradative lipase enzyme.

An astounding array of toxins isolated from bacterial pathogens assist bacteria colonization and ensure cell survival by taking over vital processes.^45^ Among the toxins, α-hemolysin (Hla) is a potent epithelial toxin that binds a variety of cells (e.g., erythrocytes, monocytes, and endothelial cells) and contributes to bacteria pathogenesis in skin infections.^46^ For instance, isogenic Hla-negative strains exhibited little or no dermo necrotic skin lesions in mice, while the wild-type USA300 strains produced dermo necrotic lesions.^47^ Conversely, in the same study, immunization with Hla-specific antisera or the nontoxic form of Hla significantly reduced the size of the skin lesions. Because the lethality of Hla is associated with its pore forming action on cells, engineered nanoparticles that function as decoys via their interactions with the toxins offer new opportunities as antitoxin formulations.^48^ In addition to the membrane-damaging activity of the hemolysins, other studies have hinted at alternative functions for these virulent factors. For instance, β-hemolysin can covalently oligomerize and precipitate DNA.^49^ Additionally, Hla mediated channel formation has drawn immense interest as a method for predominantly detecting DNA.^50^ To explore the interactions between the released DNA nanoparticles and Hla, changes in the absorbance spectra of DNA and the effect on nanoparticle size was investigated. A dose-dependent reduction in the absorbance peak of ssDNA was observed with increasing concentration of Hla, as shown in Figure 3D. For instance, the UV band at 260 nm gave an absorbance of 0.137 ± 0.005 a.u. in the presence of 8 μg/mL of the toxin compared to the DNA alone (0.1623 ± 0.0015 a.u.) or the toxin alone (0.054 ± 0.001 a.u.). Comparatively, at the lowest concentration tested (1 μg/mL), an absorbance reading of 0.155 ± 0.008 a.u. was recorded. Next, we evaluated the effect of the toxin on the size of the DNA nanoparticles using DLS. As shown in Figure 3E, in the absence of the toxin, the size of the nanoparticles was 58.08 ± 2.91 nm. A significant increase in the size of the nanoparticles (165.47 ± 20.72 nm) was observed in the presence of 8 μg/mL, confirming the strong binding affinity between the toxin and the DNA nanoparticles. These results demonstrate the potential capacity of the entrapped nanoparticles to mediate Hla toxicity by interacting with the toxin.

The pH-dependent binding of the PDM 90/5/5 formulation to bacteria was evaluated by monitoring changes in zeta potential. As shown in Figure 3F, the zeta potential of planktonic *S. aureus* was highly anionic at both physiological and acidic pH conditions (4.6–7.4). The zeta potential of the PDM 90/5/5 formulation was increasingly cationic as the pH reduced. Because of the binding of the formulation to the bacteria, a drop in the surface potential of the formulation was observed. This was more significant at lower pH conditions of 5.5 (*p* < 0.001) and 4.6 (*p* < 0.001) compared with pH 6.7 (*p* < 0.01). No difference in the surface potential of the formulation was seen at physiological pH due to the neutral surface potential of the PDM 90/575 formulation.

### 3.5 Encapsulation Efficiency and *In Vitro* Release Profile

**Figure 4:**
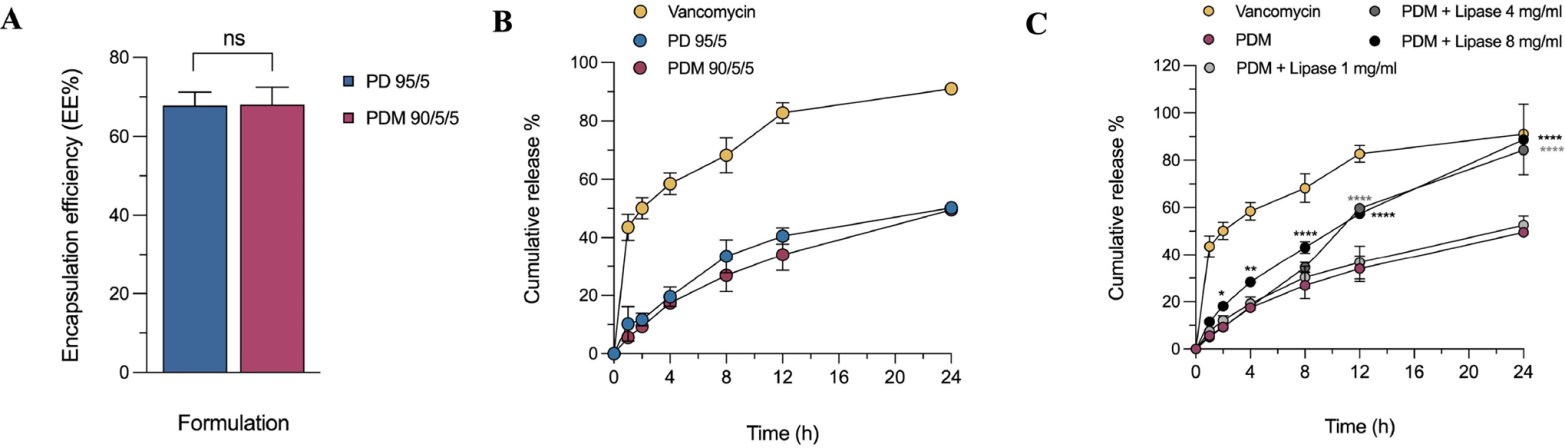
Entrapment efficiency and drug release measurements. (A) Effect of surface modification on the entrapment efficiency of vancomycin (*n* = 2). (B) *In vitro* drug release measurements of free vancomycin, PD 95/5 and PDM 90/5/5 at pH 7.4. (C) *In vitro* drug release measurements of free vancomycin, PDM 90/5/5 in the presence of different concentrations of lipase. Values based on mean ± SD, *n* = 3.

High drug entrapment is advantageous to sustain therapeutic concentrations of antimicrobials within the infection site. While the surface chemistry of nanomaterials can enhance the performance of antibacterial drugs, it is prudent to ensure that surface modification does not hamper drug encapsulation or release negatively. Therefore, to probe the effect of the surface modification on the drug entrapment, the encapsulation efficiency of vancomycin was quantified via dialysis. As shown in Figure 4A, we observed no significant difference between the entrapment of vancomycin in the PD formulation (67.8 ± 3.39%) and the PDM formulation (68.05 ± 4.45%). The observed entrapment efficiency was lesser than previous reports using neutral lipids (76.59 ± 3.44%).^15^ We propose that the reduced entrapment can be attributed to competition between DODMA and vancomycin and/or repulsion between the anionic DNA nanoparticles and PPM lipid. Nevertheless, the PDM formulation still retained a higher loading of vancomycin than the vancomycin loaded liposomes (64.64 ± 0.73%) from the study.^15^

DDS with stimuli-responsiveness can prevent premature degradation, sustain the release, and increase the local bioavailability of the drug at the desired target site.^51^ Therefore, the release behavior of vancomycin from the PDM formulation at physiological pH and in the presence of bacterial lipase was assessed using the Franz diffusion cell. As shown in Figure 4B, PD 95/5 formulation released 40.58 ± 2.85% of vancomycin within 12 h. The total drug release at 24 h was approximately 50.21 ± 1.78%. No significant difference was observed for the PDM 90/5/5 formulation (34.05 ± 5.43%) after 12 h. After 24 h, 49.57 ± 1.46% of vancomycin was release from the PDM 90/5/5 formulation, which was like the PD 95/5 formulation. Conversely, approximately 43.53 ± 4.45% and 91.06 ± 1.86% of free vancomycin was released within 1 and 24 h, respectively (Figure 4B). These results demonstrate that the zwitterionic formulations sustained the released of vancomycin over 24 h. Additionally, they provide an indication that the surface potential of the two formulations did not play a significant role in the release kinetics of the entrapped antibiotic. Toward eliminating premature drug release from formulations, other saccharides such as alginate and chitosan have been used to modify liposomal formulations.^43,44^ For instance, liposomes coated with palmitoyl dextran were fabricated for the release of hirudin. Although the formulations sustained the release of hirudin over 600 h, the authors reported a burst release of 30% within 5 h. Comparatively, the PDM 90/5/5 formulation sustained the release of vancomycin over 4 h (17.42 ± 1.91).

Next the release profile was assessed in the presence of different concentrations of lipase. As shown in Figure 4C in the presence of 1 mg/mL of the enzyme, we observed no difference in the release behavior of the PDM 90/5/5 formulation. In contrast, rapid release profiles were seen at higher lipase concentrations of 4 and 8 mg/mL. These results clearly indicate the lipase-sensitive release of vancomycin from PDM formulations in agreement with the size assessments (Figure 3C).

### 3.6 *In Vitro* Antibiofilm Activity

**Figure 5:**
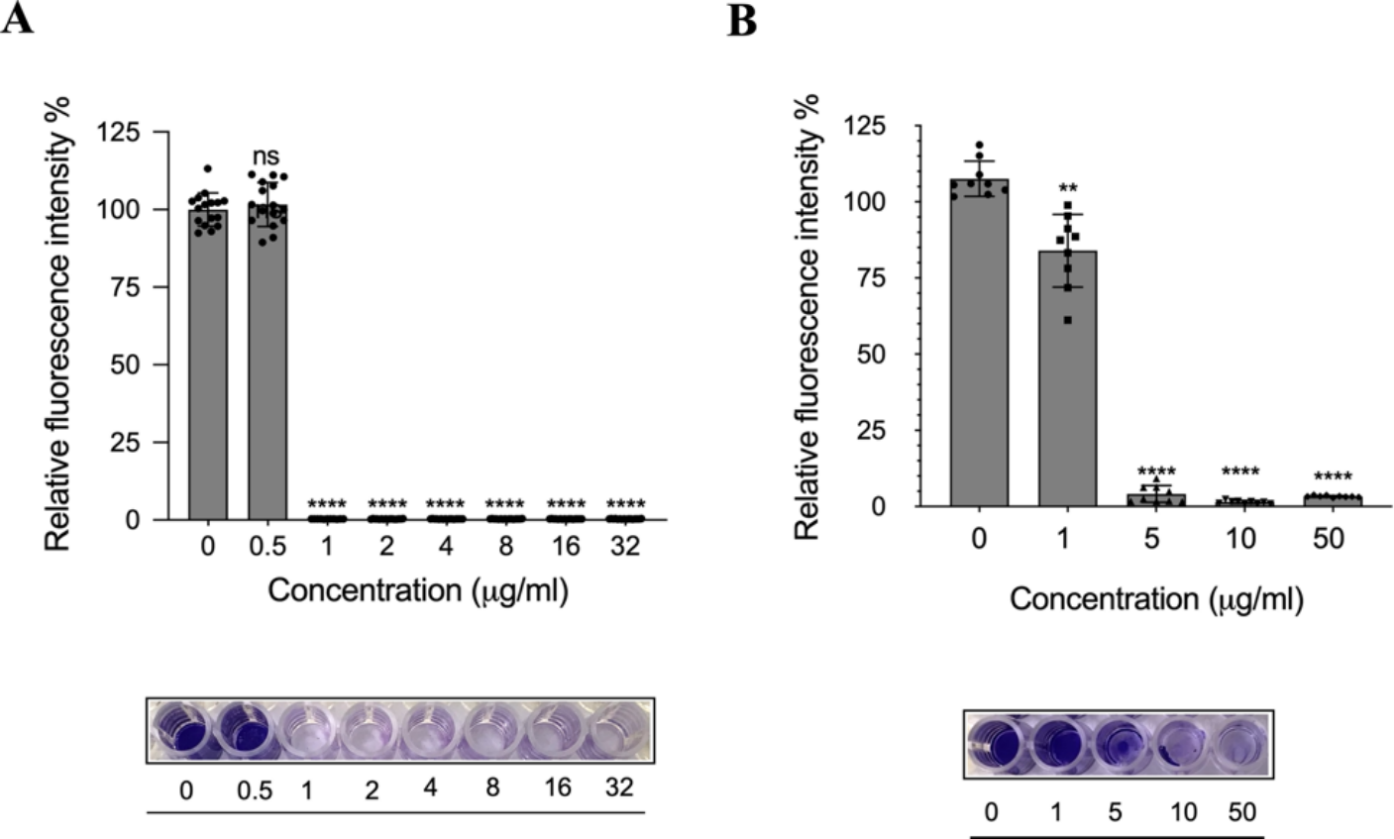
*In vitro* antibiofilm efficacy of the PDM 90/5/5 formulations. (A) Effect of the PDM 90/5/5 formulation on biofilm inhibition and crystal violet staining image. Values based on mean ± SD, *n* = 3. (B) Effect of the PDM 90/5/5 formulation on early stage biofilm eradication and crystal violet staining image. Values based on mean ± SD, *n* = 3.

Biofilm resistance is a precursor to delayed healing of chronic wounds and imposes extreme microbial tolerance to antibiotic therapy.^52^ Because of the strongly adherent nature of biofilms to surrounding tissues, physical strategies such a debridement are unable to completely remove the entire bioburden.^53^ Antibiotics represent the current choice of treatment to eradicate biofilms, but their successful accumulation within biofilms is challenging because of poor penetration, instability, and rapid degradation in the wound environment.^54^ Additionally, the poor blood circulation in chronic wounds warrants suitable delivery approaches (e.g., local administration) to overcome the limitations of systemic antibiotics.^54^ While zwitterionic lipid-based systems with multifunctional features can enable self-targeting of microbial biofilms and mediate toxicity, high potency against biofilms is essential for their clinical translation. We first evaluated the ability of the optimized formulation to prevent the attachment of bacteria by performing biofilm inhibitory assays against *S. aureus*. Using the GFP-expressing *S. aureus* ((NCTC 8325 pCM29-GFP), changes in bacterial fluorescence was monitored over 24 h. Following treatment with 0.5 μg/mL of vancomycin loaded into the zwitterionic nanoparticles, no significant reduction in biofilm formation was observed in comparison to the untreated control group (100%) (Figure 5A).

Exposure to a higher concentration of 1 μg/mL led to a significantly lower relative fluorescence intensity (0.32 ± 0.02%) of the bacteria, which demonstrates complete inhibition of the biofilm growth. To correlate the fluorescence readings to the biomass, the well-established crystal violet (CV) staining method was performed. Correspondingly, a significant reduction in the biofilm mass was observed at 1 μg/mL of the vancomycin-loaded nanoparticles and at higher concentrations (Figure 5A). For instance, at 1 μg/mL, the measured biofilm mass was ∼26.1% (as compared with 100% for untreated control group) (Figure S4A). At higher concentrations (2–8 μg/mL), we observed ∼87% reduction in the biomass following treatment with the formulations.

Next, we assessed the efficacy of the formulations against early stage biofilms grown for 6 h. As shown in Figure 5B, at 1 μg/mL of vancomycin, a significantly reduced biofilm bioburden was demonstrated with a relative fluorescence intensity of 83.98 ± 11.92%. At higher concentrations of 5 μg/mL, a further improved eradication profile with a relative fluorescence intensity of 4.05 ± 2.77% was observed. CV staining correlated the above-mentioned fluorescence findings. A drastically reduced biofilm mass of 69.8 ± 8.05% was observed when exposed to 5 μg/mL PDM 90/5/5 as shown in Figure S4B. At 50 μg/mL PDM 90/5/5, we observed ∼80% reduction in the biomass. These findings indicate that while the zwitterionic nanoparticles sustained vancomycin release, it preserved the antimicrobial activity of vancomycin when present at the targeted bacterial site.

### 3.7 Biofilm Penetration, Biocompatibility, and *Ex Vivo* Efficacy

**Figure 6:**
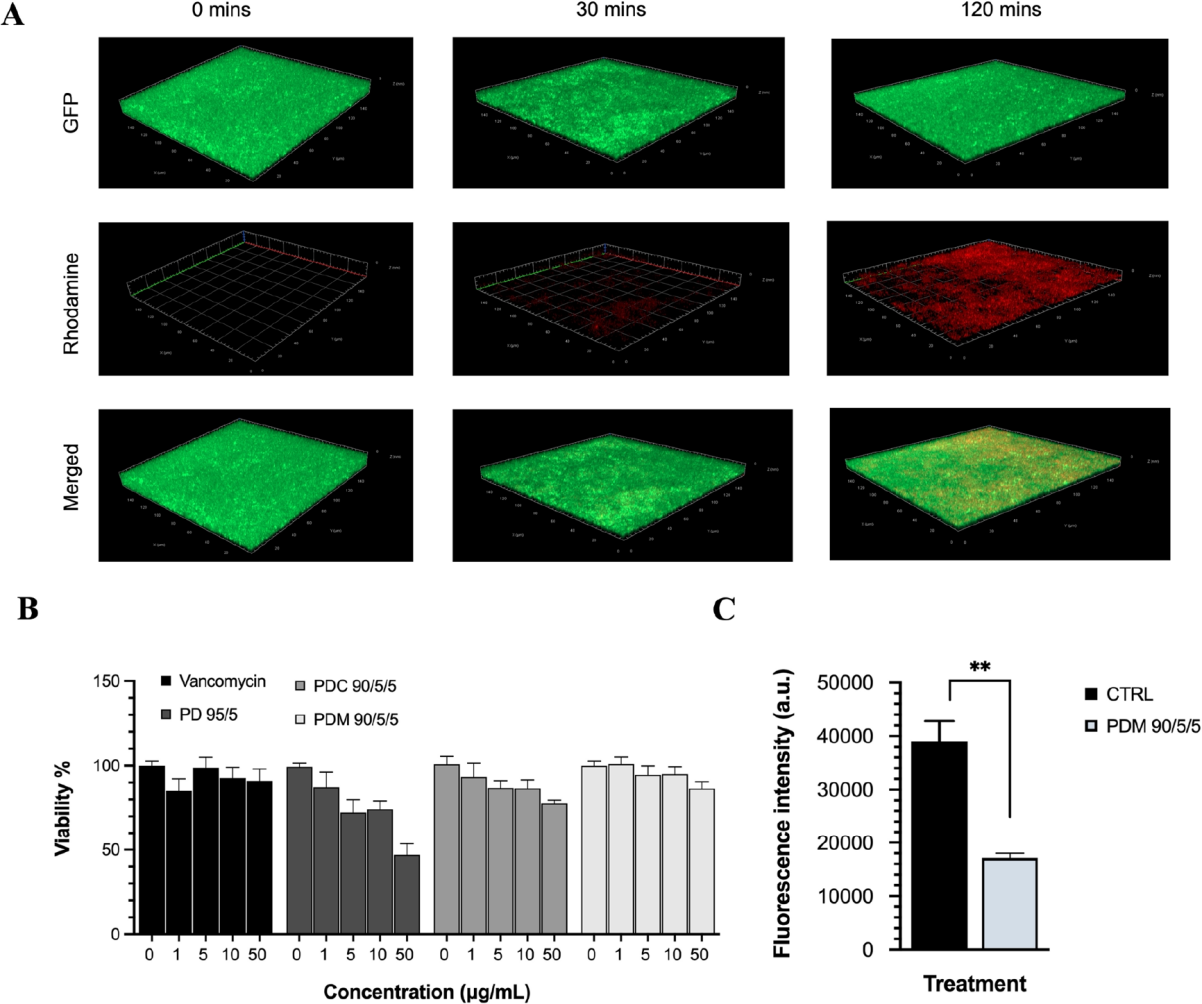
*In vitro* biofilm binding and penetration, biocompatibility, and *ex vivo* antibiofilm efficacy of the PDM 90/5/5 formulations. (A) CLSM 3D-images of GFP-labeled *S. aureus* biofilms after exposure PDM^Rho^ 90/5/5 (50 μg/mL) for 0, 30, and 120 min. (B) Cytotoxicity of free vancomycin and formulations against HaCaT cells. Values based on mean ± SD, *n* = 3. (C) Effect of the PDM 90/5/5 formulation on an *ex vivo* porcine biofilm model. Data presented as mean ± SD (*n* = 3).

Failed treatment of wound infections can often be attributed to the restricted penetration of antimicrobials. To enhance local drug accumulation within the infection site, DDS that bind and penetrate the layers of the biofilm to deliver the cargo are warranted. Thus, we investigated the penetration of the zwitterionic nanoparticles through the biofilms via confocal laser scanning microscopy. The time-dependent penetration of the rhodamine labeled formulations (PDM^Rho^ 90/5/5) was investigated within mature *S. aureus* biofilms. As shown in Figure 6A, after 30 min of exposure to PDM^Rho^ 90/5/5 (50 μg/mL), a weak red fluorescence within mature *S. aureus* biofilms and a dominant green fluorescence from the bacteria was observed.

However, after 120 min of exposure to the formulations, increased red fluorescence from rhodamine B was observed. More importantly, the merged image of the two fluorophores showed colocalization of the PDM^Rho^ 90/5/5 with the biofilm which confirms EPS penetration and bacterial uptake (within the biofilm). These results corroborate the binding affinity of the formulation with the biofilm and demonstrate rapid accumulation of the formulation in *S. aureus* biofilms within 2h.

Nonbiological pharmaceutical products can induce unwanted immune responses and/or other undesired side effects. To ascertain the biocompatibility of the optimized formulation and the effect of surface modification on toxicity, we compared the effect of free vancomycin to the vancomycin loaded formulations on keratinocyte cells (HaCaT) *in vitro*. Free vancomycin at 1 μg/mL to 50 μg/mL demonstrated negligible toxicity as shown in Figure 6B. Exposure to 50 μg/mL of the cationic PD 95/5 for 24 h resulted in significantly lower cellular viability (47,02 ± 4.03%) compared to the untreated control group (i.e., 0 μg/mL of PD 95/5). Formulations that cause reduced viability (<70%) *in vitro* indicate undesired toxicity.^55^ This implies a concentration-dependent toxicity for the PD 95/5 formulations. However, exposure to 50 μg/mL of PDC 90/5/5 resulted in a slightly improved viability of 76,28 ± 3.07%. Conversely, when the cells were exposed to PDM 90/5/5 at the same concentration, the viability exceeded 80% (i.e., 86.57 ± 3.79%), which indicates a good degree of biocompatibility toward HaCaT cells. We posit that this observation can be attributed to the modulation of the surface potential in the PDM zwitterionic (−0.02 ± 1.89 mV) formulation, compared with the highly cationic formulations; PD (+23.09 mV ± 3.46 mV) and PDC (+14.39 ± 2.30 mV) formulations. Previous studies have shown that cationic lipid-based nanoparticles exhibit greater toxicity compared with neutral or anionic nanoparticles.^25,26^ In another study, liposomes with highly cationic zeta potentials were more toxic compared with liposomes with lower zeta potentials.^56^

To further assess the translational value of the zwitterionic nanoparticles, the ability of PDM 90/5/5 formulations to eradicate mature cutaneous biofilms was investigated using an *ex vivo* porcine explant model. As shown in Figure 6C, a fluorescence intensity of 39058.7 ± 3779.1 arbitrary units (a.u.) was observed 24 h after biofilm establishment. Conversely, a significant reduction in fluorescence intensity (17124 ± 884.6) on the skin sections was observed after the PDM 90/5/5 formulation was topically applied to the mature biofilms. This resulted in a percentage fluorescence reduction of 56.16 ± 2.26% and indicates the antibiofilm capacity of the formulation within 24 h. These findings correlate with the *in vitro* results and reveal the antimicrobial potency of the PDM 90/5/5 formulation as well as demonstrates their potential as an effective delivery system in topical biofilm therapy.

## 4 Conclusion

A series of zwitterionic nanoparticles were fabricated to limit the establishment of cutaneous biofilm infections. The optimized nanocarrier (PDM 90/5/5) was loaded with antimicrobial nucleic acid nanoparticles and complexed with a neutral (90 mol % SPC), anionic (5 mol % PPM), and ionizable (5 mol % DODMA) lipid. Upon exposure to microbial pH conditions, the neutrally charged nanocarrier displayed a cationic surface potential to enhance bacteria interaction and biofilm penetration. Lipolysis of the PDM 90/5/5 formulation in response to lipases sustained the release of the carrier (to bind α-hemolysin) and vancomycin (to prevent the establishment of *S. aureus* biofilms). By manipulating the composition of the lipids, PDM 90/5/5 significantly reduced the toxicity of the cationic control and showed significant *ex vivo* potency against *S. aureus* skin infections. Altogether, this multiresponsive nanosystem is an efficient carrier for nucleic acid therapeutics and peptide drugs, and is potent against biofilm infections.

## Supporting Information Available

Size distribution of PD 95/5, PDM 90/5/5, PDC 90/5/5, PDC 85/5/10; size distribution at physiological pH for PDM 90/5/5 and PDC 90/5/5 over 24 h; effect of formulations on fluorescent LPS at physiological and acidic pH; biofilm biomass quantification after treatment with the PDM 90/5/5 formulation (PDF)
