# Supplementary material for: Biofilm Responsive Zwitterionic Antimicrobial Nanoparticles to Treat Cutaneous Infection
Source: Biomacromolecules. 2021 Dec 16;23(1):303–15. doi: 10.1021/acs.biomac.1c01274 (PMC8753600; doi:10.1021/acs.biomac.1c01274)
Supplement: Supplementary file 1 — bm1c01274_si_001.pdf [file bm1c01274_si_001.pdf]

Biofilm Responsive Zwitterionic Antimicrobial Nanoparticles to Treat Cutaneous Infection

Sybil Obuobi[1], Anna Ngoc Phung[1], Kjersti Julin[2], Mona Johannessen[2], Nataša Škalko-Basnet[1]*

[1]Drug Transport and Delivery Research Group, Department of Pharmacy, UIT The Arctic University of Norway, Tromsø, Norway.

[2] Host Microbe Interaction research group, Department of Medical Biology, UIT The Arctic University of Norway, Tromsø, Norway.

RESULTS

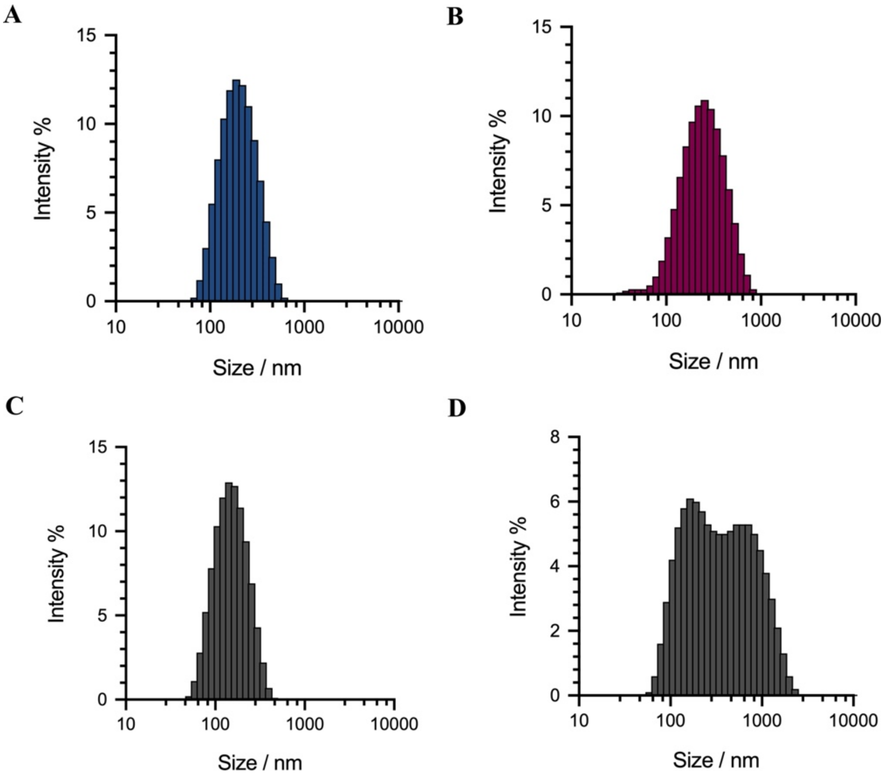

Figure S1. Size distribution of the A) PD 95/5 formulation. B) PDM 90/5/5 formulation. C) PDC 90/5/5 formulation. D) PDC 85/5/10 formulation.

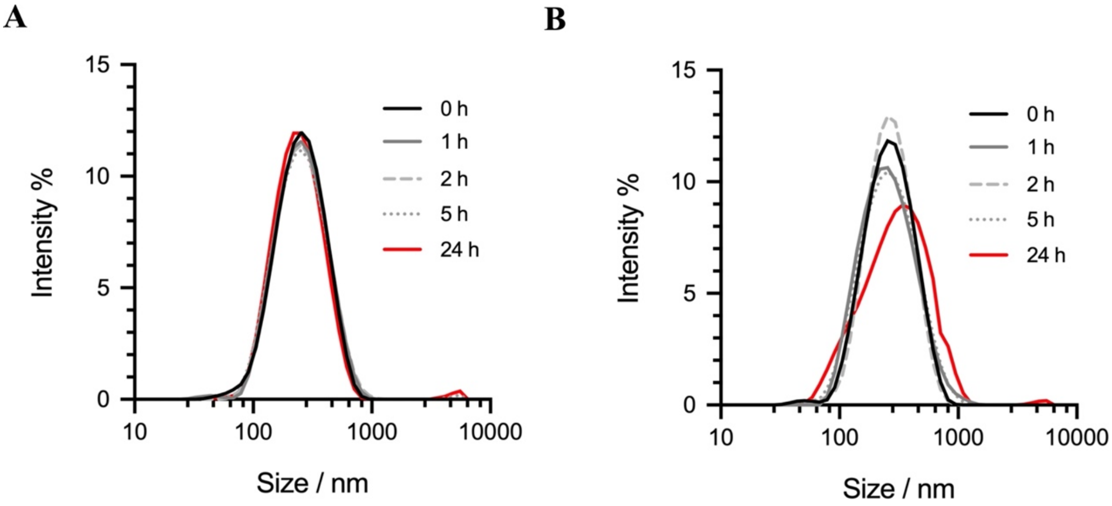

Figure S2. Size distribution of the A) PDM 90/5/5 formulation at pH 7.4 over 24 h. B) Size distribution of the PDC 90/5/5 formulation at pH 7.4 over 24 h.

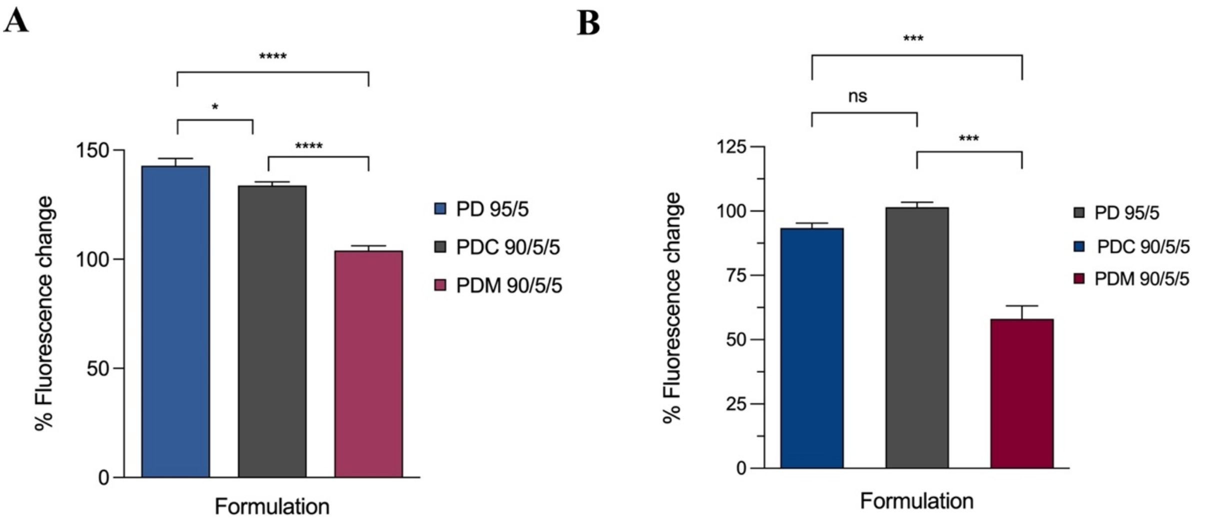

Figure S3. Effect of the antimicrobial formulations on AlexaFluor 594 LPS. A) Effect of the antimicrobial formulations on AlexaFluor 594 LPS at physiological pH. B Effect of the antimicrobial formulations on Alexa 594 LPS at acidic pH (pH 5.5).

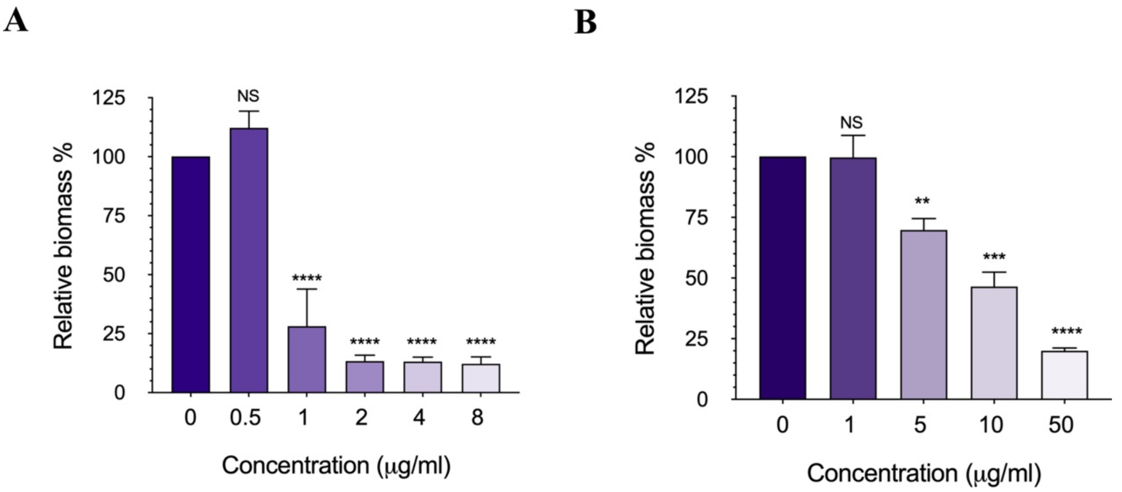

Figure S4. Crystal Violet staining. A) Biofilm biomass quantification for the inhibitory effect of PDM 90/5/5. B) Biofilm biomass quantification of early-stage biofilm formation after treatment with the PDM 90/5/5 formulation. Values based on mean ± SD, n=3.
